# The Cytotoxic and Apoptotic Effects of the Brown Algae *Colpomenia sinuosa* are Mediated by the Generation of Reactive Oxygen Species

**DOI:** 10.3390/molecules25081993

**Published:** 2020-04-24

**Authors:** Reem Al Monla, Zeina Dassouki, Achraf Kouzayha, Yahya Salma, Hala Gali-Muhtasib, Hiba Mawlawi

**Affiliations:** 1 Laboratory of Applied Biotechnology (LBA3B), AZM Center for Research in Biotechnology and its Applications, Doctoral School for Sciences and Technology, Lebanese University, Tripoli 1300, Lebanon; reem.monla95@gmail.com (R.A.M.); achraf.kouzayha@ul.edu.lb (A.K.); yahyasalma@ul.edu.lb (Y.S.); 2 Department of Biology and Department of Anatomy, Cell Biology and Physiological Sciences, American University of Beirut, Beirut 1107 2020, Lebanon; 3 Center for Drug Discovery, American University of Beirut, Riad El Solh, Beirut 1107 2020, Lebanon

**Keywords:** brown algae, cytotoxic, apoptosis, cell cycle, reactive oxygen species, human colon cancer

## Abstract

Brown algae are a novel resource of biogenic molecules, however few studies have been conducted in the Mediterranean to assess the cytotoxic mechanisms of algal-derived compounds. This study focuses on the antineoplastic activity of extracts from non-investigated algae of the Lebanese coast, *Colpomenia sinuosa*. Extracts’ antineoplastic activities were evaluated by MTT and trypan blue on different tumorigenic cells. Results indicated that the most potent extract was obtained by soxhlet using dichloromethane:methanol solvent (DM soxhlet) against HCT-116. Wound healing assay confirmed that this extract decreased the migration potential of HCT-116 cells with minimal effects on non-tumorigenic cells. It also induced an increase in the subG1 population as determined by flow cytometry. Western blot analysis demonstrated that apoptosis in treated HCT-116 cells was induced via upregulation of p21 protein and downregulation of the anti-apoptotic Bcl 2, which led to caspases activation. The latter, catalyzes the degradation of PARP-1, and thus suppresses cancer proliferation. Morphological alterations, further confirmed apoptosis. A strong pro-oxidant activity evidenced by the enhanced generation of reactive oxygen species (ROS) was observed in HCT-116 treated cells. Interestingly, a strong antioxidant effectively blocked effect induced by the extract. These results indicate that *C. sinuosa* is a source of bioactive compounds possessing pro-apoptotic and anti-migratory efficacy.

## 1. Introduction

Carcinogenesis is a multistep process characterized by several hallmarks, including enhanced proliferative signaling, evasion of growth suppressors, resistance to cell death, as well as activation of invasion and metastasis [1]. The etiology of cancer is multifactorial, and involves many external and internal factors that can act together or in sequence to cause the disease [2]. It is noteworthy to point that cancer cells with increased oxidative stress are more sensitive to damage of proteins, apoptosis and degradation by further ROS triggered exogenous agents [3].

Apoptosis is a process of programmed cell death that maintains tissue and cellular homeostasis. It is characterized by several morphological features. It can be activated by two main signaling pathways through cascades of caspases: the extrinsic, in which cell death receptors act as initiation point of the apoptotic process, and the intrinsic pathway, where stimuli act on an intracellular target and the mitochondria plays a major role [4]. The Bcl 2 family, is a key regulator of mitochondrial outer membrane permeabilization and can suppress the release of cytochrome c and other factors that are important for the activation of caspases and hence apoptosis [5]. Two types of caspases are critically involved in apoptosis. The first type is the initiator caspases, while the second type includes the executioner caspase 3 [6]. The latter, catalyzes PARP-1 cleavage, which is overexpressed in various carcinomas. Caspase 3 cell death is also associated with upregulation of p53 protein and the p53-regulated gene p21 [7]. Upon activation, p21 upregulates p53 and induces the blocking process of the cell cycle at the G1 and/or S phase [8]. One of the common characteristics of cancers is their resistance to cell death stimuli. Therefore, compounds that activate apoptosis have the potential to be implicated in the pharmaceutical industry [9].

Chemotherapy is a commonly used therapeutic modality in cancer, involving various drugs acting on both malignant and healthy cells, and causing lethal side effects. Thus, new approaches to improve tolerance and reduce sequelae of cancer chemotherapy are urgently needed. Biomolecules extracted from natural marine organisms are well known to inhibit the carcinogenic pathway in malignant cell lines with minimal toxic and side effects [10]. 

Macroalgae have a wide and dynamic taxonomy. The three main seaweed phyla are Rhodophyta, Ochrophyta (brown algae), and Chlorophyta [11]. They are rich in bioactive materials including polysaccharides, phycocyanins, terpenes, and fucosterols. In vivo and in vitro examinations emphasized the role of crude extracts from diverse brown seaweeds as a promising anticancer candidates [12,13]. Multiple species of brown algae have been previously investigated and were found to have great biological potentials [13,14,15,16].

Brown seaweeds synthesize biogenic molecules with pharmaceutical potential to counteract environmental stress [17]. Also, this type of algae has been indicated to contain higher antioxidant potential than red and green seaweeds [18]. Amongst the most interesting activities is their cytotoxic potential against a range of malignant tumors [19]. 

The Lebanese coast is a great area for investigating a wide range of macroalgal biomass, due to several factors including high biodiversity, warm seasons, and proximity to the Suez Canal which makes it a center for exotic biota in the Mediterranean region [20,21]. Most of studies done over the Lebanese algae against cancer are very limited and unrecognizable as they mostly depend on colorimetric methods without studying the mechanism of action of the crude extracts or polysaccharides [22,23,24].

*Colpomenia sinuosa (C. sinuosa)* is an edible brown algae, belonging to the order of Scytosiphonales, and the family of Scytosiphonaceae [25,26]. It is commonly known as brown bag weed [27]. Morphology of C. sinuosa was described decades ago; it bears a sessile thallus which is attached by a broad base; it is leathery and hollow, globular when young, becoming expanded and folded as it grows older [28]. Historically, it was found that this species occurs abundantly in the Mediterranean [29]. 

*C. sinuosa* is used as human food, due to the presence of antioxidant properties, a good source of phenols, vitamins, folic and amino acids [30]. *C. sinuosa* is high in ash content, and is rich in unsaturated fatty acids, fucosterols, stigmasterols, palmitic acid and the amino acid lysine [28,29,30,31,32,33]. Iron levels in *C. sinuosa* species of the Mediterranean sea waters (Beirut) is three times higher than *C. sinuosa* located in other regions [34]. The presence of the free monosaccharides glucuronic acid, galactose and xylose in *C. sinuosa* did not differ significantly among seasons of collection [35]. Fucoxanthin isolated from this seaweed significantly increased percentage of death in breast cancer cells [36]. Moreover, fucogalactoglucan isolated from *C. peregrina* showed potent cytotoxic effects against human cervical cancer cell line, as it also exhibited immunomodulatory potentials on both cellular and molecular levels [37]. *C. sinuosa* is the first marine species where the cytotoxic colpol, a dibromide phenylbutane metabolite, was found and discovered [38]. *C. sinuosa* collected from the Persian gulf did not show any significant cytotoxicity against a range of cancer cell lines [39]. On the contrary, extracts of *C. sinuosa*, collected from Taiwan, induced apoptosis in human leukemia cells through the generation of ROS [40]. However, *C. sinuosa* cytotoxic mechanisms of action, specifically against colon cancer, are poorly studied and most previous studies were limited to cytotoxicity tests only. In this sense, this study is the first to report the cytotoxic activity of *C. sinuosa* extracts from the Lebanese Mediterranean coast against four types of solid tumors.

## 2. Results and Discussion

### 2.1. C. sinuosa Organic and Aqueous Extracts Inhibited Cancer Cell Viability 

In our recent work, the proximal analysis of Lebanese *C. sinuosa* revealed high levels of total carbohydrate and ash content, with moderate total protein and lipid content [41]. In this study, nine extracts isolated from *C. sinuosa* were tested for their antiproliferative properties against four human cancer cell lines: Cervical cancer (HeLa), breast cancer (MCF-7), and two colon cancer cell lines (HCT-116 and HT-29).

**Figure 1:**
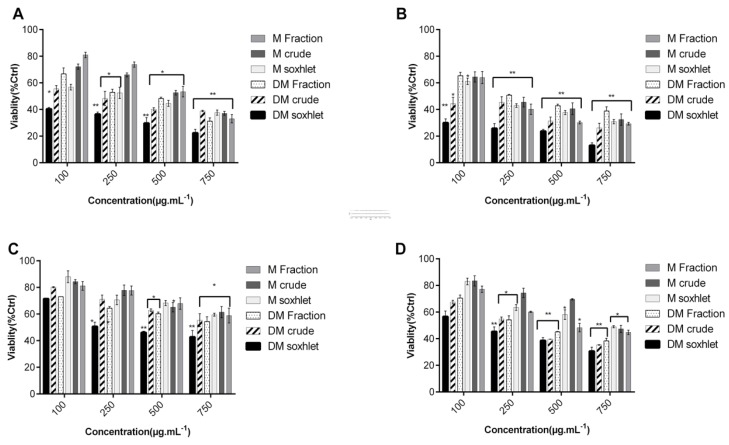
Cytotoxicity of *C. sinuosa* organic extracts on colon cancer by MTT assay. Cells were seeded in 96-well plates and treated subsequently with 100–750 µg·mL^−1^ of extracts: (**A**,**B**) Effect on HCT-116 post 24 h treatment and 48 h treatment, respectively; (**C**,**D**) Effect on HT-29 cell line post 24 and 48 h treatments, respectively. Results are reported as the mean ± SD from three independent experiments (n = 3). * p < 0.05 and ** p < 0.01 with respect to control.

**Figure 2:**
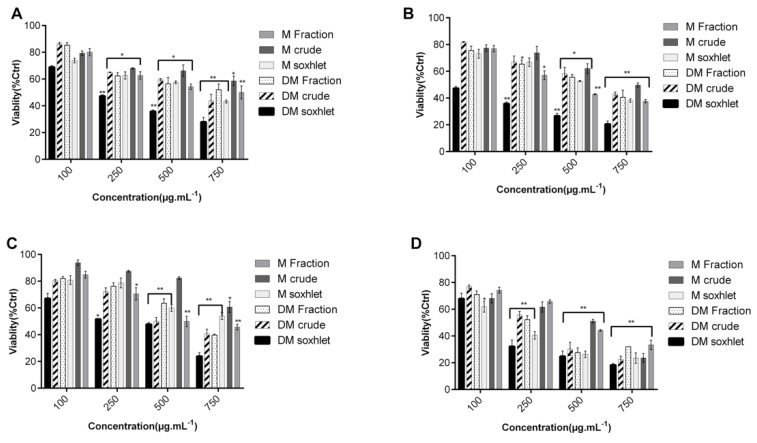
Cytotoxic activity of different concentrations of *C. sinuosa* extracts on HeLa and MCF-7 cell lines determined by MTT assay: (**A**,**B**) Effect on HeLa at 24 and 48 h; (**C**,**D**) Effect on MCF-7 at 24 and 48 h. Results are reported as the mean ± SD from three independent experiments (n = 3). * *p < 0.05* and ** p < 0.01 with respect to control.

Upon treatment with various extracts of *C. sinuosa* for 24 or 48 h, all organic extracts (DM and M extracts), induced a significant decrease in cell viability in a dose- and time-dependent manner (Figure 1 and Figure 2). These extracts were found to be more potent against HCT-116 cell line. We believe that cytotoxic molecules present in the *C. sinuosa* were more soluble in mildly non polar organic solvents. These results are in accordance with many other studies revealing the significant cytotoxic activity of organic extracts against a range of cancer cell lines [24,42,43].

Interestingly, extraction at high temperatures, significantly increased the inhibitory effect of DM and M extracts on cell viability (at 100 μg·mL^−1^, the viability of HCT-116 cells treated with DM soxhlet was 36.6 ± 3.6%, lower than that of DM crude 44.37 ± 4.7%) (Figure 1B).

Indeed, DM soxhlet extract exhibited the most potent effect against all solid tumor cell lines and had the lowest IC_50_ value (Supplementary Tables S1 and S2). In addition; it was noted that the IC_50_ of DM soxhlet against HT-29 at 24 h (379 ± 1.19 µg·mL^−1^) was significantly higher than that obtained against HCT-116 (42.57 ± 1.64 µg·mL^−1^). This highlights an important aspect of *C. sinuosa* DM extracts, which is their tumor specificity that enables them to differentially target cancer cells. 

On the other hand, Aq extracts showed low cytotoxic effects in comparison to the extracts obtained from M and DM extraction (Supplementary Figures S1 and S2). This is in parallel to many other studies revealing the low efficacy of aqueous extracts against different tumors [44,45]. These results were confirmed by trypan blue, where the extracts showed a similar pattern of cytotoxicity in comparison to MTT (Supplementary Materials, Figure S3).

In a previous study, we showed that DM soxhlet extract of this algae are rich in phenols (27.7 ± 0.2 mg GAE.g-1 dried sample). DM soxhlet extract had a higher phenolic content than all methanol extracts [41]. Despite their high phenolic content, Aq extracts showed low anticancer properties. This can be attributed to many reasons, including the polarity, size, and lipophilicity of the molecules. It is well known that moderately polar molecules are able to passively diffuse across the cell membrane, in contrast to larger highly-polar metabolites that exhibit lower efficacy and cell permeability [46,47]. Based on these reasons, we believe that due to the high molecular weight polysaccharides present in the aqueous extracts and their high hydrophilicity, the bio adsorption by cells, and hence the cytotoxic activity against these tested cancer cells was reduced. 

**Figure 3:**
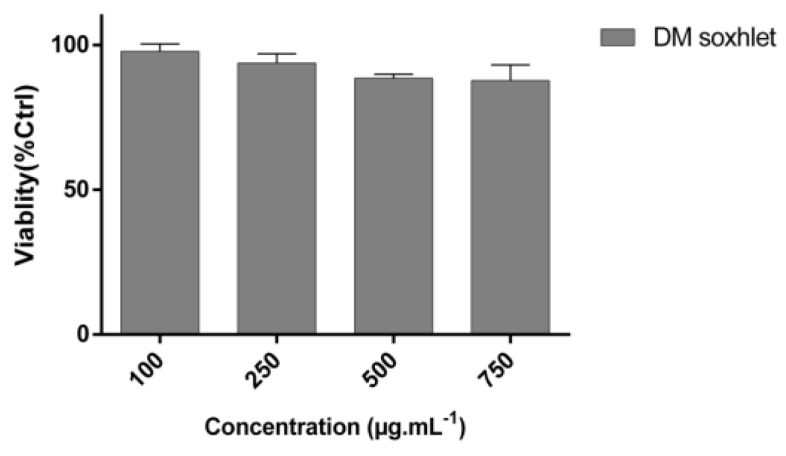
Minimal cytotoxic effects of dichloromethane:methanol solvent (DM soxhlet) extract against the NCM460 non-tumorigenic human colon cell line. Results by MTT are reported as the mean ± SD from three independent experiments (n = 3).

MTT test was also done to test the cytotoxic activity of the most potent extract, DM soxhlet, against NCM460 non-tumorigenic colon cell line (Figure 3). There was a non-significant decrease in the percentage of cell viability in response to the extracts, even at maximum concentrations of 750 µg·mL^−1^, where the percentage of viable cells remained high (87.67 ± 5.44%) (Figure 3).

### 2.2. DM Soxhlet Extract Significantly Decreased the Migration of HCT-116 Cancer Cell Lines 

**Figure 4:**
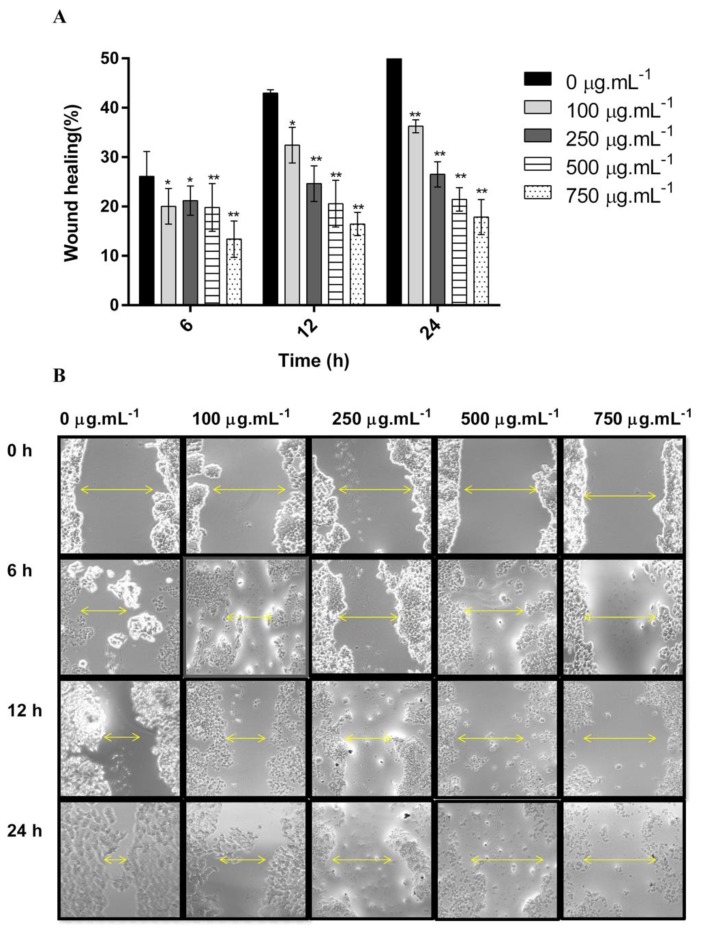
In vitro anti-migratory potentials of DM soxhlet extract on HCT-116 cell line migration analyzed by wound healing assay. “Wound” was created by a straight line scratch across the cancer cells monolayer: (**A**) Percentage of cell-covered area at 6, 12, and 24 h over 0 h as mean ± SD; (**B**) Representative images of two independent experiments done in triplicate. Significant differences are indicated as * p < 0.05 and ** p < 0.01 with respect to control.

In this study, we reported for the first time the potent anti-migratory activity of the DM soxhlet extract obtained from *C. sinuosa*. The anti-migration properties of the DM soxhlet extract were evaluated by the wound healing assay against the HCT-116 cell line. Results showed that DM soxhlet extract significantly reduced the gap closure rates in a dose- and time-dependent manner (Figure 4A,B). 

All treated conditions revealed a significantly lower rate in gap closure in comparison to control (57.25 ± 1.73%). At 24 h, the wound healing percentages were: 36.25 ± 1.32, 26.5 ± 2.51, 21.43 ± 2.37, and 17.85 ± 3.55% at 100, 250, 500, and 750 µg·mL^−1^, respectively (Figure 4B). This decrease in the percentage of migration rate in the treated cells relative to control further proves the potential of this extract to inhibit colon cancer cellular migration and invasion in vitro.

### 2.3. C. sinuosa Extract Induced A SubG1 Increase in HCT-116 Cancer Cells

**Figure 5:**
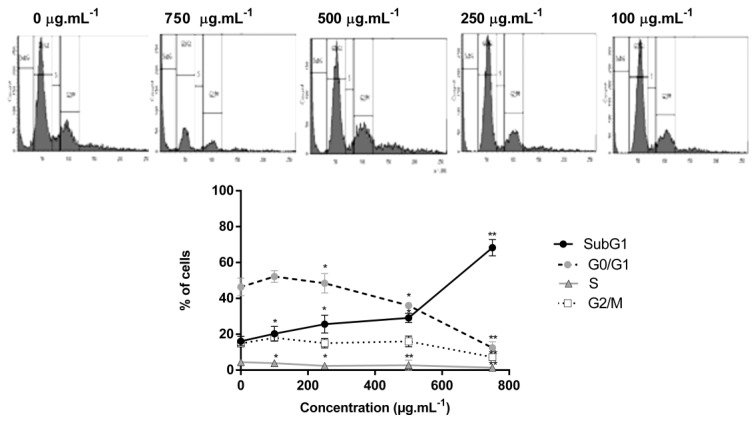
Flow cytometry analysis confirms that DM soxhlet extract of *C. sinuosa* increases the subG1 population in colon cancer cells at 24 h. Significant differences vs. control cells, comparing cell cycle phases (SubG1, G0/G1, S, G2/M), are indicated by * p < 0.05 and by ** p < 0.01. Data values represent the mean ± SD (n = 2).

To investigate the basis of the cytotoxic properties, cell cycle analysis was performed using flow cytometry (Figure 5).

Significant changes in cell cycle were determined in the presence of DM soxhlet extract. Untreated cells presented a typical cytogram of a diploid cell population. Whereas treated cells at 750 µg·mL^−1^ accumulated significantly in the subG1 phase (more than 3 fold increase) in comparison to control, and the number of cells in G0/G1 phase decreased drastically upon treatment to reach 12.45 ± 3.23% compared to 46.4 ± 4.87% in control. The number of cells in S and G2/M phases decreased in a similar manner upon treatment (Figure 5).

Deregulation of cell cycle is closely related with apoptosis. The significant increase of cell population at the subG1 phase suggests induction of apoptosis or necrosis in the DM soxhlet treated group.

### 2.4. DM Soxhlet Extract Induced Apoptosis in HCT-116 Cell Line through the Regulation of Apoptotic Related Proteins

**Figure 6:**
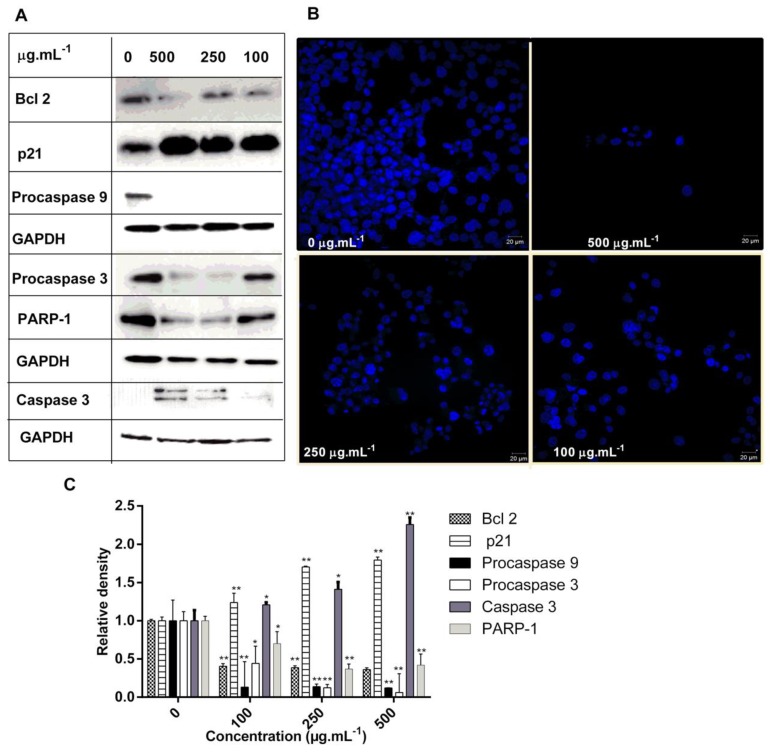
DM soxlet promotes apoptosis in colon carcinoma cells: (**A**) Representative Western blot analysis showing a significant increase of p21 and decrease in the levels of Bcl 2, procaspases, and PARP-1, along with caspase 3 activation. Expression of GAPDH was used as an internal control; (**B**) DAPI micrographs of HCT-116 cells treated with DM soxhlet extract at different concentrations (500, 250, 100, and 0 µg·mL^−1^). Nuclei were counterstained with DAPI (blue) at 40× magnification Scale bars = 20 μm; (**C)** Quantification of the western blots using image J analysis * p < 0.05 and ** p < 0.01.

Apoptosis is a strictly regulated pathway activated to restrain the survival of abnormal cells that become deregulated in tumorigenesis [48]. Treatment of HCT-116 cells with DM soxhlet extract at 100, 250, and 500 μg.mL^−1^, dose-dependently increased the expression of p21 protein, and caused the cleavage of caspase 3 in addition to decreasing the expression of procaspase 9, 3, PARP-1, and Bcl 2 (Figure 6A,C). 

The upregulation of p21 clearly affects cell cycle progression in G1 phase and cell proliferation which is well established upon cell cycle analysis of treated cells. Upon treatment at 500 µg·mL^−1^, the expression of p21 was found to be increased by 1.79-fold. Collectively, DM soxhlet showed a significant apoptosis-inducing effect via the mitochondrial apoptotic pathway by decreasing the expression of Bcl 2 and upregulating the expression of caspase 3 (Figure 6A,C). In the present study, PARP-1 expression decreased dose dependently upon treatment. Also, we showed that accelerated apoptotic cell death diminished the protein expression levels of procaspase 9, and procaspase 3 upon treatment (Figure 6A,C). Taken together, these results indicate that the DM soxhlet extract induces apoptosis in HCT-116 cancer cells, by activating caspase-mediated apoptotic signaling (Figure 6A,C). 

The in vitro studies above supported the antineoplastic effects and provided insights into the key molecules associated with its activity on HCT-116 cell line. Expression levels of Bcl 2, proteins that are essential for cancer cell survival and play a crucial role in protecting mitochondria from cellular malfunctions during apoptosis [49], was significantly reduced in response to treatment. 

Furthermore, morphological changes of HCT-116 were investigated using DAPI staining, where the cells were imaged under a confocal laser scanning microscope. Morphological features revealed that the toxicity of DM soxhlet extract on HCT-116 cancer cells caused alterations in the size, shape, and volume of cells (Figure 6B). Control cells showed typical features of well proliferative cancer cells. Indeed, membrane blebbing, condensed and fragmented nuclei were the features of the cells treated at 500 µg·mL^−1^. Most cells appeared to be dead with tiny nuclei when treated with DM soxhlet extract, especially at high concentrations (Figure 6B). Apoptotic features affirmed by DAPI staining, confirm that this extract induces apoptosis and inhibits colon cancer cell proliferation.

### 2.5. ROS Mediate the Cytostatic Effects of the DM Soxhlet Extract in HCT116 Cancer Cells 

Inducing excess ROS levels by redox modulation is a strategy to selectively kill cancer cells without causing significant damage to normal cells [50]. Many natural molecules play dual roles in the cellular state: First, as an antioxidant that scavenge free radicals. Second, as a pro-oxidant which may drive the Fenton reaction, leading to formation of ROS [51].

**Figure 7:**
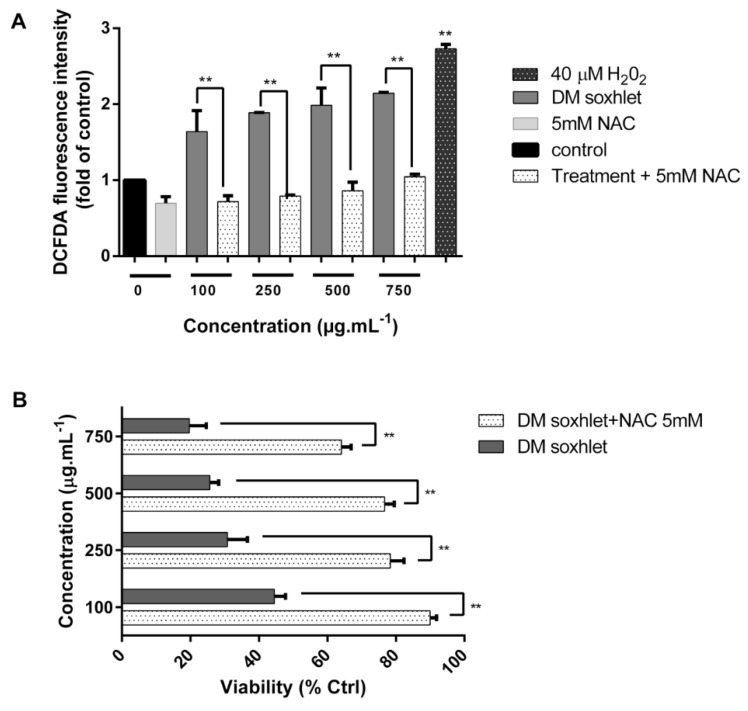
DM soxhlet extract triggers an increase in DCFDA fluorescence intensity (ROS production) in HCT-116 colon cancer cells: (**A**) Quantitative analysis of DCFDA intensity upon treatment with increasing concentrations of extract; (**B**) pretreatment with the ROS inhibitor NAC for 1 h prior to the 24 h incubation with DM soxhlet extract, further confirmed the role of ROS in the extract cytotoxic activity. Data values represent the mean ± SD (n = 3). * p < 0.05 and ** p < 0.01.

After establishing the inhibitory effect of the DM soxhlet treatment on HCT-116 cell line viability and cell cycle regulation, we measured ROS levels by CM-H2DCFDA assay in treated cells to determine if the extract activated intracellular ROS generation. As shown in Figure 6A, the level of ROS significantly increased in a dose-dependent manner after treatment. In fact, the fluorescence intensity in DCF-positive treated cells was significantly increased compared to the respective untreated HCT-116 cells (Figure 7A). 

Quantitative analysis showed that increasing concentrations of the DM soxhlet extract significantly elevated ROS levels by 1.63 folds at 100 µg·mL^−1^, to reach 2.145 folds at 750 µg·mL^−1^, in comparison to control untreated cells. Moreover, pretreatment with 5 mM NAC significantly reduced ROS generation after DM soxhlet extract incubation, and completely blocked the effect on ROS induction at all tested concentrations (Figure 7A). This decrease in DCFDA fluorescence levels is due to NAC antioxidant and inhibitory effect on ROS generation induced by the extract. To further examine whether ROS are responsible for the antineoplastic effects of the DM soxhlet extract, we pretreated cells with the strong antioxidant NAC (5 mM for 1 h) to block ROS generation in HCT-116 cells and assessed cell viability. Interestingly, NAC pretreatment significantly blocked the cytotoxic effects of the DM soxhlet extract in HCT-116 cells (Figure 7B).

Henceforth, DM soxhlet extract resulted in a ROS-mediated apoptosis in HCT-116 cells, due to the accumulation of intracellular ROS, which was later abrogated upon NAC pretreatment. The ROS-mediated effects were also confirmed by the fact that pretreatment with NAC significantly attenuated the cytotoxic effects of the DM soxhlet extract by MTT. In a study conducted in Taiwan, ethyl acetate extract *of C. sinuosa* also induced ROS generation and apoptosis in leukemic an effect that was suppressed by NAC [40]. Thus, our results are in agreement with published data from Taiwan although the extraction methods and solvents used are different in these two studies.

## 3. Materials and Methods 

### 3.1. Marine Algal Material 

*C. sinuosa* samples were collected in July 2018, from the North Lebanese coast of the Mediterranean, specifically from Al Qalamoun, at a depth of 3–5 m. Fresh seaweeds were rinsed with tap water and polished to remove associated epiphytes, salt, sand, microorganisms and other materials. Then, it was air dried in a shady place at room temperature (20–27 °C), then ground to a fine powder. A herbarium voucher is kept in preservation at the Lebanese University, *Doctoral School of Science and Technology*. Morphology of the collected algae is shown in Supplementary Figure S4.

Preparation of *C. sinuosa* extracts: Four types of solvent extractions were followed using three different solvents. These include cyclohexane, dichloromethane: methanol (DM, 1:1), methanol (M), and finally water or aqueous (Aq). For crude maceration, 30 g of sample were macerated for three days in 300 mL in solvent at room temperature in an orbital shaker. Soxhlet extraction was done using the same solvents but with soxhlet extractor at elevated temperatures for 6 h. For sequential maceration, samples were sequentially extracted by soaking in various solvent systems starting with cyclohexane, DM (1:1, *v*/*v*), M, and Aq. Each solvent extraction process was conducted for three days (Supplementary Figure S5). The extracts were then concentrated using a rotary evaporator. For the water extracts, the samples were lyophilized. 

### 3.2. Cell Lines and Cell Culture

Colon cancer cells (HT-29 and HCT-116), breast cancer (MCF-7) and human cervical cancer cells (HeLa), were purchased from the American Type Culture Collection (ATCC). Cells were cultured in DMEM at 37 °C in a humidified atmosphere of 5% CO_2_ and 95% air. Media was supplemented with 1% Penicillin Streptomycin (100 U·mL^−1^) and 10% heat-inactivated fetal bovine serum (FBS).

### 3.3. Cell Viability Assay

The cell growth assay is an MTT-based method that measures the ability of metabolically active cells to convert tetrazolium salt into a blue formazan product [39]. Cells were seeded overnight in a 96 well plate at a density of 10^4^, cells were then treated with various concentrations of extracts (100–750 µg·mL^−1^). Different extracts of *C. sinuosa* were used for treatment: DM, M, and Aq (crude, fraction, and soxhlet). After 24 and 48 h treatment, cells were incubated with MTT for 2 h at 37 °C in the dark. The mean absorbance values of three experiments were expressed as percentage of viability relative to the control untreated cells. The most potent extract was subjected to MTT assay to test its cytotoxicity on NCM460 cell line. NCM460 is a non-tumorigenic epithelial colon cell line, and is derived from normal colon mucosa [52]. For certain experiments, HCT-116 cells were preincubated with 5 mM NAC (NAC, Sigma) for 1 h and then treated with algae extract for 23 h. Absorbances were measured by the ELISA microplate reader at 570 nm.

### 3.4. Trypan Blue Test 

Cancer cells (MCF-7, HeLa, HCT-116, and HT-29) were seeded at a density of 5 × 10^4^ in a 24-well plate. Cells were later treated with different concentrations of *C. sinuosa* extracts. Based on MTT data, only four organic extracts were tested: DM and M extracts (crude and soxhlet). After 24 and 48 h, cells were washed with PBS, harvested and stained with trypan blue (0.4%). The number of viable (unstained) versus dead (stained) cells were counted using a hemocytometer using a light microscope. 

### 3.5. Wound-Healing Migration Assay 

HCT-116 cells were seeded in a 24 well plate and cultured in growing medium for 24 h. At confluency, a scratch wound was applied with a sterile 200 µL tip. Cell debris was washed twice with PBS. Cells were then incubated with algal extract at different concentrations. Images of the wounds were taken immediately after wounding (0 h). After 6, 12, and 24 h of incubation, the wound widths were measured again in the same area. The percentage of wound-healing migration was calculated by considering the wound closure at 0 h as 100%. Images were recorded using a digital camera coupled to an optical microscope. Surface area was analyzed by image J analysis program. Areas where the cells were completely dead and floating due to cytotoxicity were omitted from the analysis. 

### 3.6. Cell Cycle Analysis 

Dead and living cells were collected 24 h after treatment with DM soxhlet extract. The pellets were washed with ice cold PBS, fixed with 70% ice-cold ethanol and stored at −20 °C overnight. Cells were then washed twice with PBS and incubated with 200 µg·mL^−1^ RNAse A for 1 h at 37 °C, before staining with 0.625 µg·mL^−1^ of PI for 30 min. The fluorescence intensity was measured by flow cytometry using a fluorescence activated cell sorter (FACSAria, Beckton Dickinson, Franklin, USA) and analyzed using Cell Quest.

### 3.7. DAPI Staining 

Morphological changes of treated cells were investigated under a confocal laser scanning microscope (ZEISS LSM710, Germany), using DAPI staining. Briefly, the cells were treated with DM soxhlet extract at different concentrations (500, 250, 100 µg·mL^−1^). After 12 h, cells were washed with ice cold 1X PBS, fixed with methanol, and stored overnight at –20 °C. Cells were washed twice, then stained with fluoroshield mounting medium with DAPI, in dark condition. Morphological changes were investigated under a confocal laser scanning microscope.

### 3.8. Western Blot Analysis 

Cells were cultured in a 60 mm dish and incubated for 24 h. After 16 h of exposure to DM soxhlet extract at designated concentrations, cells were collected by trypsinization and washed with cold PBS. Collected cells were lysed by 2X laemmli buffer. The lysates were centrifuged at 20,000 g for 30 min. Proteins were quantified using the DC Protein Assay. Lysates were separated by SDS-PAGE and then transferred on nitrocellulose membranes. Membranes were then blocked with 5% skim milk for 2 h and then probed with appropriate dilutions of the primary antibodies according to the manufacturer’s directions. Membranes were washed for 10 min (3×), with tris buffer saline buffer. Then incubated with the appropriate secondary antibodies for 1 h. Protein–antibody complexes were detected by a chemiluminescence. Equal amounts of loaded proteins were verified by probing for GAPDH (Abcam, ab9484). 

### 3.9. Quantitative ROS Determination

Generation of intracellular ROS in cancer cells provide a unique opportunity to eliminate cancer via the activation of various ROS-induced cell death pathways [53]. The generation of intracellular ROS was monitored quantitatively using CM-H2DCFDA. This method is based on the formation of highly fluorescent 2′,7′-dichlorofluorescein (DCF) from non-fluorescent CM-H2DCFDA by ROS [54]. HCT-116 cells were seeded in 96-well culture plates and allowed to adhere overnight in a CO_2_ incubator at 37 °C. HCT-116 cells were pre-incubated with 5 mM NAC for 1 h in certain conditions. Hydrogen peroxide (20 µM H_2_O_2_), was used as a positive control to induce ROS production. Cells were treated with different concentrations of DM soxhlet extract, and incubated for 4 h at 37 °C. At the end of the respective treatment period, cells were incubated with 20 µM working solution of CM-H2DCFDA dye in a serum free and phenol red free medium at 37 °C for 30 min. Fluorescence intensity was detected using a microplate fluorometer (TriStar^2^, Berthold, USA) at an excitation wavelength of 485 nm and an emission wavelength of 528 nm.

### 3.10. Statistical Analysis

All statistical analysis (t-test and one-way ANOVA) were performed using GraphPad Prism 7 (version 7.0, GraphPad Software Inc., La Jolla, CA, USA). Probability values below 0.05 (* *p* < 0.05) were considered significant and values below 0.01 (** *p* < 0.01) were considered highly significant. Quantitative data are expressed as means ± SD from the indicated set of experiments.

## 4. Conclusions

In summary, this study provided evidence for the potential of different extracts of the brown macroalgae *C. sinuosa,* collected from the North Lebanese coast, to inhibit the viability of a range of solid tumors. The organic DM soxhet extract revealed a significant cytotoxic activity against HCT-116 human colon cancer cells, with minimal cytotoxic effects on NCM460 non-tumorigenic colon cells. This extract further inhibited the migration of HCT-116 cells. The mechanistic approaches revealed that DM soxhlet extract induced apoptosis and morphological changes in colon cancer cells via the generation of ROS. Apoptosis induction occurred by the upregulation of p21 and the activation of caspases 3 and 9, followed by PARP-1 cleavage. Based on the obtained data, DM soxhlet extract could be considered as a valuable source of selective cytotoxic molecules. In comparison to other extracts obtained from *C. sinuosa*, DM soxhlet extract was the most potent, as the solvent used was able to extract the cytotoxic molecules of mild polarity, in addition to its high phenolic content. Studies are underway to elucidate the chemical structure of the most bioactive compound present in this extract for potential combination with standard clinical drugs. Considering the nutritional value of seaweeds, the DM soxhlet extract could also be proposed as a dietary supplement in combination with conventional therapy.

## Supplementary Materials

The following are available online. Figure S1: Effect of Aqueous extracts obtained from *C. sinuosa* on HCT-116 (A,B at 24 and 48 h respectively) and HT29 (C,D at 24 and 48 h respectively) by MTT assay, Figure S2: Cytotoxic effect of aqueous extracts against MCF7 at 24 and 48 h (A,B respectively) and HeLa at 24 and 48 h (C and D respectively) by MTT assay, Figure S3: Trypan blue assay confirms the cytotoxic potentials of organic extracts against A. HeLa, B. HCT-116, C. HT-29 and D. MCF7 cell line post 24 h treatment, Figure S4: Morphology of *C. sinuosa* collected from the North Lebanese coast, Figure S5 Extraction process of *C. sinuosa*, Table S1: IC_50_ of DM, M and Aq extracts of the Lebanese *C. sinuosa* on different cell lines at 24 h, Table S2. IC_50_ of DM, M and Aq extracts of the Lebanese *C. sinuosa* on different cell lines at 48 h.
