# Supplementary material for: The Cytotoxic and Apoptotic Effects of the Brown Algae Colpomenia sinuosa are Mediated by the Generation of Reactive Oxygen Species
Source: Molecules. 2020 Apr 24;25(8):1993. doi: 10.3390/molecules25081993 (PMC7221617; doi:10.3390/molecules25081993)
Supplement: Supplementary file 1 [file molecules-25-01993-s001.pdf]

The cytotoxic and apoptotic effects of the brown algae *Colpomenia sinuosa* are mediated by the generation of reactive oxygen species

Table S1. IC_{50} of DM, M and Aq extracts of the Lebanese *C. sinuosa* on different cell lines at 24h

Cell line	*C. sinuosa* extract	IC50(µg/ml)		
		Crude	fraction	soxhlet
HCT-116	M	478.9±1.203	481.1±1.164	248.4±1.76
	DM	190.3±1.08	255±1.35	42.57±1.65
	Aq	6739±1.42	6734±1.14	8708±1.23
HT-29	M	23961±1.4	2014±1.61	1383±3.27
	DM	1379±1.02	1266±1.61	261.5±3.27
	Aq	1655±1.29	4053±1.9	1589±1.21
HELA	M	1778±1.41	673.9±1.14	614.7±1.29
	DM	620±1.16	660.4±1.2	121±1.79
	Aq	2378±1.61	4207±1.25	2630±1.33
MCF7	M	1109±1.29	573.3±1.08	951.1±1.29
	DM	646.8±1.242	672.2±1.3	277.3±1.27
	Aq	2962±1.78	2766±1.33	1978±1.48

Table S2. IC_{50} of DM, M and Aq extracts of the Lebanese *C. sinuosa* on different cell lines at 48h

Cell line	*C. sinuosa* extract	IC50(µg/ml)		
		Crude	fraction	soxhlet
HCT-116	M	233.2±1.11	179.9±1.15	192.3±1.12
	DM	79.3±1.8	386.4±1.05	12.76±3.54
	Aq	1630±1.34	1091±1.44	2617±2.15
HT-29	M	1383±1.48	615.1±1.21	691.8±1.19
	DM	301±1.48	234.6±1.21	104.6±1.69
	Aq	1419±1.72	2609±1.57	1397±1.17
HELA	M	904±1.3	377.2±1.06	496.5±1.2
	DM	612.7±1.11	547.9±1.16	24.27±1.48
	Aq	1788±1.23	1707±1.29	1896±1.96
MCF7	M	341.8±1.33	394.2±1.077	172.1±1.08
	DM	274.5±1.05	246.4±1.11	166±1.14
	Aq	1425±1.88	1643±1.4	1566±1.55

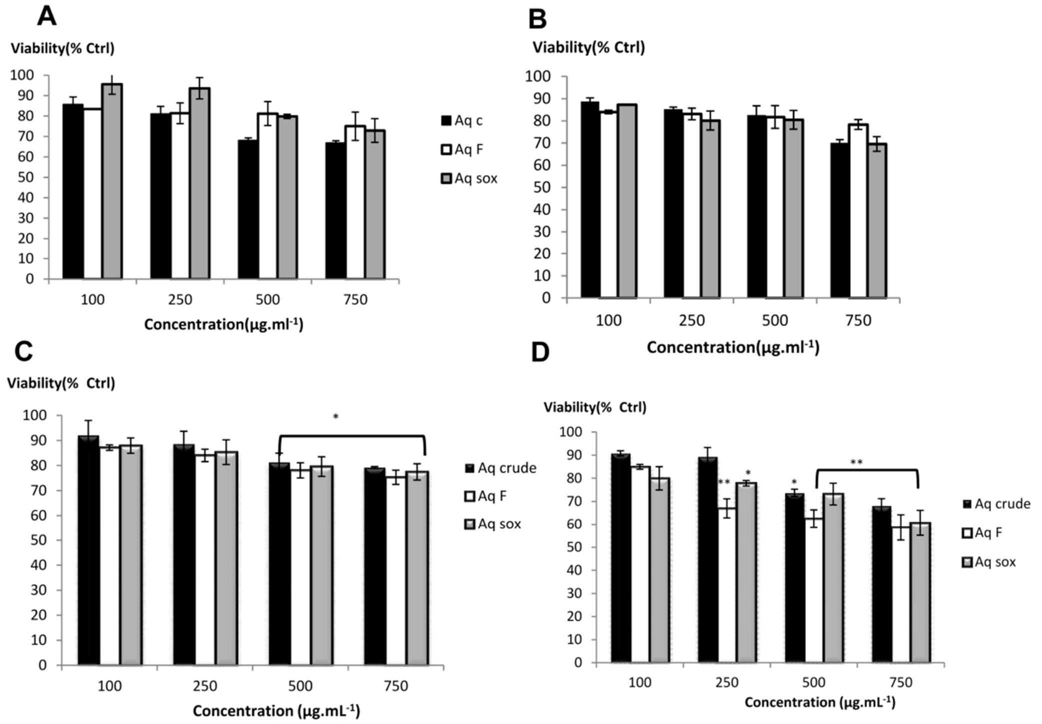

Figure S1 Effect of Aqueous extracts obtained from *C. sinuosa* on HC-T116 (A,B at 24 and 48 h respectively) and HT29 (C,D at 24 and 48h respectively) by MTT assay.

Aq F : Aqueous fraction

Aq sox: Aqueous extract obtained by soxhlet

Aq crude: Crude aqueous extract

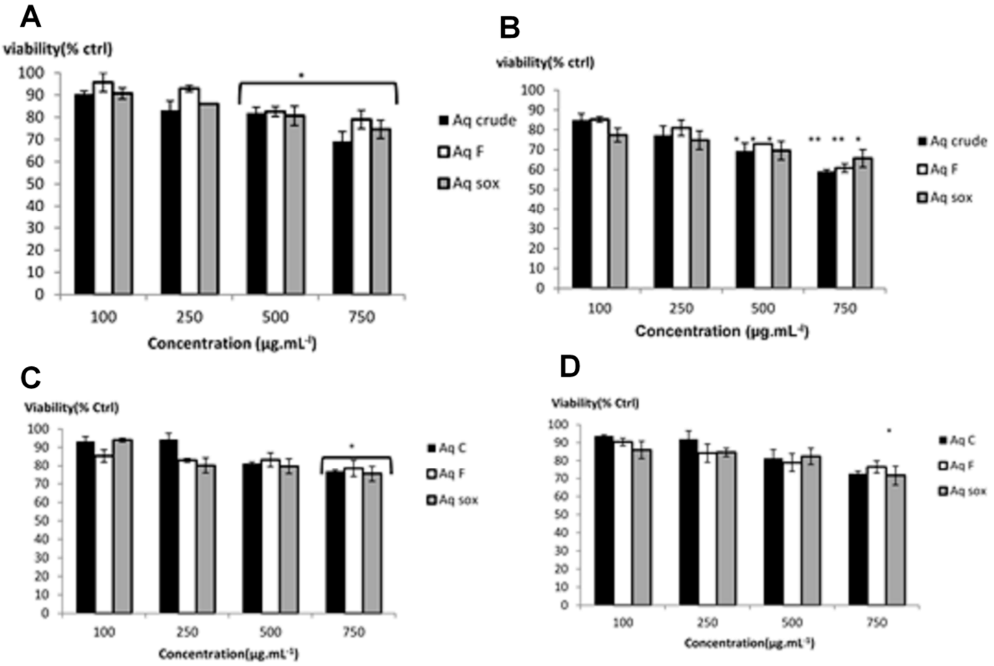

Figure S2 Cytotoxic effect of aqueous extracts against MCF7 at 24 and 48 h (A,B respectively) and HeLa at 24 and 48h (C and D respectively) by MTT assay.

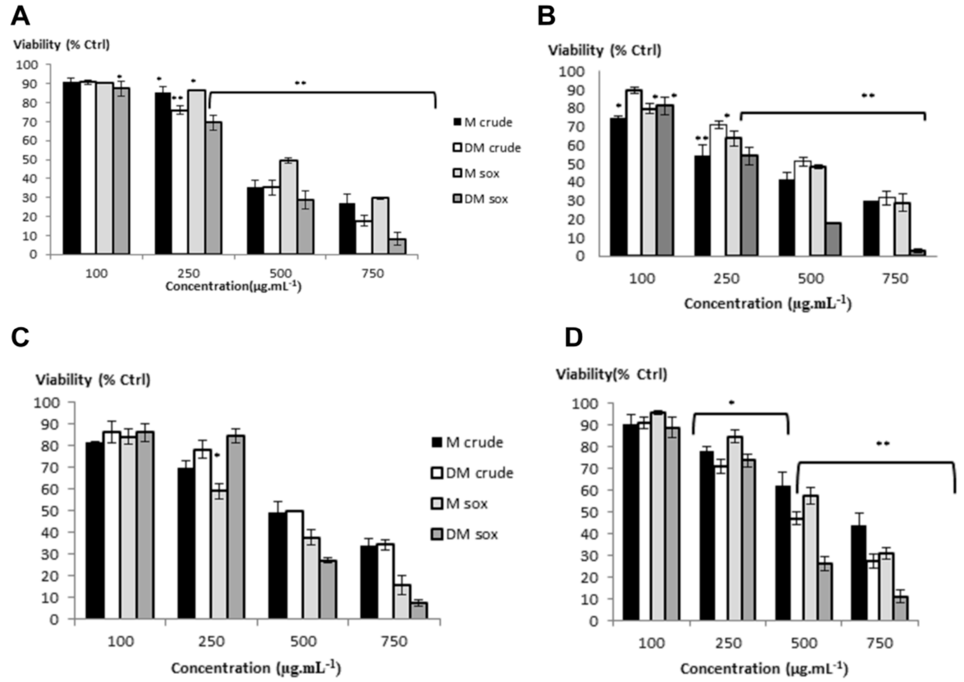

Figure S3 Trypan blue assay confirms the cytotoxic potentials of organic extracts against A. HeLa, B. HCT-116, C. HT-29 and D, MCF7 cell line post 24 h treatment.

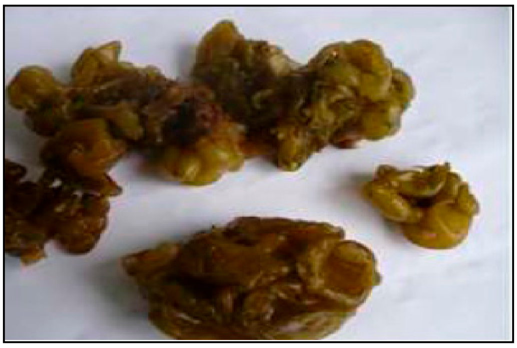

Figure S4 Morphology of *C. sinuosa* collected from the North Lebanese coast

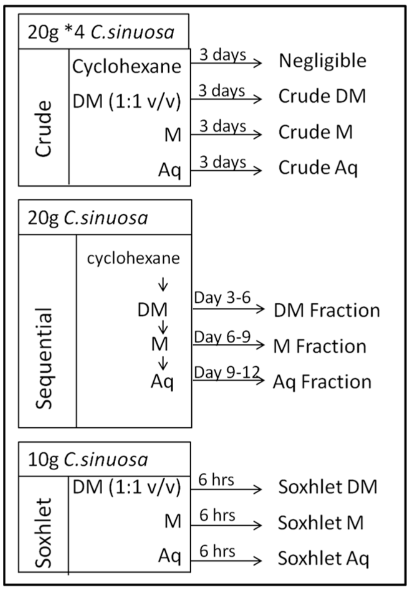

Figure S5 Extraction process of *C. sinuosa*
